# Comprehensive analysis of cuproptosis-related lncRNAs in immune infiltration and prognosis in hepatocellular carcinoma

**DOI:** 10.1186/s12859-022-05091-1

**Published:** 2023-01-03

**Authors:** Chunhua Liu, Simin Wu, Liying Lai, Jinyu Liu, Zhaofu Guo, Zegen Ye, Xiang Chen

**Affiliations:** 1 Rehabilitation Center, The Second Affiliated Hospital of Wenzhou Medical University, 108 Xueyuan West Road, Wenzhou, Zhejiang China; 2 Department of Cancer Rehabilitation, Lishui Hospital of Traditional Chinese Medicine Affiliated to the Zhejiang University of Chinese Medicine, Lishui, Zhejiang China

**Keywords:** Cuproptosis-related lncRNAs, Hepatocellular carcinoma, Immune infiltration, Tumor immune microenvironment, Drug therapy

## Abstract

**Background:**

Being among the most common malignancies worldwide, hepatocellular carcinoma (HCC) accounting for the third cause of cancer mortality. The regulation of cell death is the most crucial step in tumor progression and has become a crucial target for nearly all therapeutic options. Cuproptosis, a copper-induced cell death, was recently reported in Science. However, its primary function in carcinogenesis is still unclear.

**Methods:**

Cuproptosis-related lncRNAs significantly associated with overall survival (OS) were screened by stepwise univariate Cox regression. The signature of cuproptosis-related lncRNAs for HCC prognosis was constructed by the LASSO algorithm and multivariate Cox regression. Further Kaplan–Meier analysis, proportional hazards model, and ROC analysis were performed. Functional annotation was performed using gene set enrichment analysis (GSEA). The relationship between prognostic cuproptosis-related lncRNAs and HCC prognosis was further explored by GEPIA(http://gepia.cancer-pku.cn/) online analysis tool. Finally, we used the ESTIMATE and XCELL algorithms to estimate stromal and immune cells in tumor tissue and cast each sample to infer the underlying mechanism of cuproptosis-related lncRNAs in the tumor immune microenvironment (TIME) of HCC patients.

**Results:**

Four cuproptosis-related lncRNAs were used to construct a prognostic lncRNA signature, which was an independent factor in predicting OS in HCC patients. Kaplan–Meier curves showed significant differences in survival rates between risk subgroups (*p* = 0.002). At the same time, we found that the expression levels of most immune checkpoint genes increased with increasing risk scores. Tumorigenesis and immunological-related pathways were primarily enhanced in the high-risk group, as determined by GSEA. The results of drug sensitivity analysis showed that compared with patients in the high-risk group, the IC50 values of erlotinib and lapatinib were lower in patients in the low-risk group, while the opposite was true for sunitinib, paclitaxel, gemcitabine, and imatinib. We also found that elevated *AL133243.2* expression was significantly associated with worse OS and disease-free survival (DFS), more advanced T stage and higher tumor grade, and reduced immune cell infiltration, suggesting that HCC patients with low *AL133243.2* expression in tumor tissues may have a better response to immunotherapy.

**Conclusion:**

Collectively, the cuproptosis-associated lncRNA signature can serve as an independent predictor to guide individual treatment strategies. Furthermore, *AL133243.2* is a promising marker for predicting immunotherapy response in HCC patients. This data may facilitate further exploration of more effective immunotherapy strategies for HCC.

**Supplementary Information:**

The online version contains supplementary material available at 10.1186/s12859-022-05091-1.

## Background

Liver cancer is among the highest prevalent existing malignancy, leading to approximately 662,000 deaths each year, and its prevalence and death rates are rising year by year globally [1, 2]. As the primary histological sub kind of liver cancer, hepatocellular carcinoma (HCC) denotes about 75% of all instances [3]. Currently, HCC is typically treated with liver resection, liver transplantation, thermal ablation, trans-arterial chemoembolization (TACE), hepatic arterial infusion chemotherapy (HAIC), personalized treatment, and immunotherapy [4, 5]. The function of protein-coding genes in HCC etiology was the subject of substantial studies for many years. Treatments for HCC have been continuously refined, but its overall prognosis remains to be suboptimal [6, 7]. Therefore, seeking suitable predictive indicators and possible treatment for HCC is crucial for a more optimal prognosis.

Copper is a trace metal that is necessary for life. Maintaining an appropriate quantity of copper performs a key part in maintaining body activities and homeostasis. Copper deficiency leads to impaired copper-binding enzyme functionality, while copper accumulation leads to cell death [8]. Peter et al. recently proved that copper ion, not copper ionophore, is cell toxic [9]. Cuproptosis is an entirely new form of cell death different from any recognized forms of death, such as apoptosis, siderosis, and necrosis [9]. During tumor metastasis, copper plays a primary role as an important catalytic cofactor for multiple biological functions within the extracellular matrix (ECM) [10]. Research has proved that cuproptosis influences the immune microenvironment of various tumors, thus affecting the effect of treatment and prognosis of patients, such as soft tissue sarcoma and melanoma [11, 12]. Cuproptosis's involvement in the genesis, progression, and fate of HCC is far from certain. In addition, as cuproptosis is a lately discovered sort of regulated cell death (RCD), its potential as an immunotherapy target deserves additional exploration.

LncRNA is a non-coding RNA with over 200 nucleotides in length. The latest research has revealed that lncRNA has significant involvement in cancer pathogenesis, involving epigenetics, cell cycle, and cell regulations [13, 14]. Along with the introduction of contemporary high-throughput sequencing techniques, more lncRNA functions have been annotated. LncRNAs such as lymphocytic leukemia 1 (DLEU1) are associated with breast cancer and up-regulate CKAP2 expression to promote breast cancer malignancy via serving as a co-activator of HIF-1α [15]. Through controlling numerous epigenetic pathways like m6A methylation, ferroptosis, and autophagy, lncRNAs may have a role in HCC development, relapse, and immunotherapy responsiveness, as proved in earlier research [16–18]. Nevertheless, research on lncRNAs linked to cuproptosis in HCC is still scarce. Furthermore, the predictive precision of lncRNAs that are related to curproptosis and their association with the tumor immune microenvironment (TIME) in HCC is still unknown. Consequently, this study aims to explore cuproptosis-related lncRNAs in HCC and to comprehend the importance of cuproptosis-related lncRNAs in TIME and prognosis that focuses on the signaling pathways and molecular pathways implicated in the cuproptosis activity in HCC and can offer unique insights for patients with HCC hoping for immunotherapy.

In the present research, we developed a predicting signature according to cuproptosis-related lncRNAs and then evaluated their functional enrichment, immune cell infiltration, chemotherapy medication susceptibility, and immune checkpoint inhibitors. Clinicians now have an effective, feasible, and statistical strategy for estimating HCC patient survival and developing individualized therapeutic options.

## Materials and methods

### Data source and cuproptosis-related lncRNAs

The mRNA expression datasets and clinical information of HCC, comprising age, gender, tumorous grade, pathological stage, and AJCC-TNM, were acquired through the Cancer Genome Atlas (TCGA) data (https://portal.gdc.cancer.gov/). To identify lncRNAs as well as mRNAs, annotation sets for GRCH38 long noncoding RNAs were also acquired through GENCODE (https://www.gencodegenes.org/human/). Transcriptome expression data were normalized using the "limma" package and obtained with the "TCGAbiolinks" package. For normalizing the transcriptome data, the log2 (FPKM+1) transformation was utilized. Next, using the perl language, we obtained 16,876 lncRNAs according to the Ensemble ids of the normalized gene symbols. In addition, the expression matrix of 19 cuproptosis-related genes was acquired through TCGA-LIHC, following the literature (Additional file 1: Table S1). Pearson correlation test was further conducted to identify cuproptosis-related lncRNAs following the filter criteria (|R|≥ 0.4 and *p* value < 0.001). As per the National Cancer Institute's December 2015 standards (https://cancergenome.nih.gov/publications/publication guidelines), no ethical approval is needed for our research.

### Construction of the cuproptosis-related lncRNA predictive signature

Random assignment was used to allocate 371 HCC subjects to either a training or testing group at a 1:1 ratio using the “caret” package. Using a combination of univariate Cox regression and Least Absolute Shrinkage and Selection Operator (LASSO) Cox regression studies, using the “glmnet” R package, a signature of lncRNAs associated with cuproptosis was generated in the training set [19]. The formula applied to calculate predictive signature risk scores was the following. Risk score = $\sum_{i=1}^{n}(Coefi \times xi)$, where Coef stands for the coefficient value, and x stands for the expression value of specified cuproptosis-related lncRNAs. A Coef value greater than zero means increased risk. Using the 'time-ROC' package, the prediction performance of the prognostic model for overall survival (OS) was determined. Kaplan–Meier (K–M) test was used to compare the OS of the training group using the R packages "survival" and "surminer".

For further investigation of the predicting efficacy of the signature of cuproptosis-related lncRNAs, the testing group and general patients were validated. The training group, the testing group, and every subject were all categorized into either high- or low-risk groups, respectively, based on the median risk score. Using the K–M method, the OS of patients in the high- and low-risk groups in the testing group and in the overall cohort was compared. On the basis of risk scores and survival statistics, using the 'pheatmap' package, we plotted survival risk curves for patients in high and low risk groups. On the basis of each patient's risk score and its clinical characteristics, using the "survival" package, we assessed whether the risk score model could be used as an independent prognostic factor for HCC patients by univariate and multivariate Cox regression analysis. Risk ratios were also calculated for these variables. Furthermore, on the basis of each patient's risk score, the “survival” and “survminer” packages were used to assess whether the risk score model was influenced by the patient's clinical characteristics.

To further explore the potential mechanism of cuproptosis in HCC, we analyzed the differences in immune infiltration and immune function between high and low risk groups, and further analyzed the relationship between immune infiltration and risk score. Based on the LIHC patient gene symbol, we measured each sample's stromal score, immune score, and estimate score using the “limma” and “estimate” packages [20]. Next, according to the risk grouping of each patient, the "limma" and "ggpubr" packages were used to analyze the differences of these parameters between the two groups. Using ssGSEA and GSVA, differences between the two groups were determined for 29 immune markers and immune-related functions per sample by the Cell-type Identification by Estimating Relative Subsets of RNA Transcripts (CIBERSORT) algorithm [21, 22].

Meanwhile, through the "corrplot", "ggpubr" and "ggExtra" packages, we analyzed the relationship between immune checkpoint inhibitory genes and risk scores. In addition, the differences between high and low risk groups were analyzed by the "limma", "reshape2", "ggplot2" and "ggpubr" packages.

### Construction and validation of a predictive nomogram

Studies have shown that Nomogram can accurately calculate the survival rate of tumor patients, and has great application value for clinical formulation of individualized treatment strategies [23]. For predicting OS in HCC patients, a predictive nomogram comprised of the cuproptosis-related lncRNA risk score and clinical parameters was developed through “rms” and “regplot” packages. The created nomogram was utilized to forecast the 1, 3, and 5-year OS, then to contrast the actual OS. The "survminer" package was used to plot the nomogram-predicted survival and the actual prognosis. The reliability of the prediction was determined using the "time-ROC" package.

### Prediction analysis of cuproptosis-related lncRNAs risk signature

For identifying the four lncRNAs expression that related to cuproptosis in the model and the connection between their expression and the outcome of HCC, we retrieved the expression, OS, and disease-free survival (DFS) maps of HCC tissues from the Gene Expression Profile Interactive Analysis (GEPIA) data (http://gepia.cancer-pku.cn/). Furmore, we assessed their expression in various clinical traits using the “limma” and “ggpubr” packages. According to the expression levels of the four cuproptosis-related lncRNAs in the samples, we divided all samples into high and low expression groups according to the median value of the expression levels. According to the immune infiltration score and gene expression level of each sample, the differences in TME between the two groups were analyzed using the "limma" and "ggpubr" packages, the difference of 29 immune markers and immune-related functions between the two groups was analyzed using the "reshape2", "ggpubr" and "ggExtra" packages. Finally, the expression differences of immune checkpoint genes between high and low-expression groups were also analyzed.

In addition, the "limma" package was employed to identify the differentially expressed genes (DEGs) between the two groupings according to the metrics: P < 0.05 and |log2(fold change)|> 1. "ClusterProfiler", "org.hss.eg.db", "enrichment", "DOSE", "ComplexHeatmap", "RColorBrewer", "dplyr", "circlize", in addition, the "ggpubr" package is used for pathways and functions Enrichment studies, including Gene Ontology (GO) biological processes, Kyoto Encyclopedia of Genes and Genomes (KEGG) pathways and visualization of their results [24, 25]. At last, GSEA was carried out to detect tumor hallmarks. Gene Set Enrichment Analysis (GSEA) 4.1.0 was employed to conduct the analysis (http://www.broad.mit.edu/gsea/). For GSEA, the number and type of permutations were set at “1000” and “no Collapse”/“phenotype”, respectively; the gene list ordering mode was set as “descending”; the gene list sorting mode was set as “real”; the metric for ranking genes was set as “Signal2Noise”. False discovery rate (FDR) < 0.05 was deemed to be the statistical significance threshold.

### The role of the predictive signature in predicting the clinical treatment response

For investigating the impact of predictive factors on estimating the responsiveness to LIHC therapy, we determined the half-maximal inhibitory concentration (IC50) of widely administered chemotherapeutic medications in the medical therapy of LIHC by using the R package “pRRophetic” [26]. Wilcoxon signed-rank testing was performed to contrast IC50 results between groups with high- and low-risk.

### Statistical analysis

Each statistical calculation was conducted employing R (Version. 4.0.2). PERL (version 5.32.1.1, https:// strawberryperl.com/) was used for gene name translation, lncRNA identification, data-file expression, and phenotyping. In order to compare the expression patterns of cuproptosis-related lncRNAs in healthy and cancerous tissues, the Wilcoxon testing was utilized. Univariate Cox regression was utilized to examine the correlation between cuproptosis-related lncRNAs and OS, while multivariate Cox was utilized to monitor cuproptosis-related lncRNAs to develop a predicting signature. The Kaplan–Meier technique as well as the logarithmic rank testing were utilized to contrast the OS of high- and low-risk subject groups. With univariate and multivariate Cox regression analysis, significant prognostic variables were detected. The "survivalROC" program was employed for developing receiver operating characteristic (ROC) curves and for estimating the area under curve (AUC) results. For ssGSEA, the "GSVA" program was utilized. The filtering conditions for all the results were: *p* < 0.05(“*”), *p* < 0.01(“**”) and *p* < 0.001(“***”).

## Results

### Identification of cuproptosis-related lncRNAs in TGCA-LIHC

**Fig. 1:**
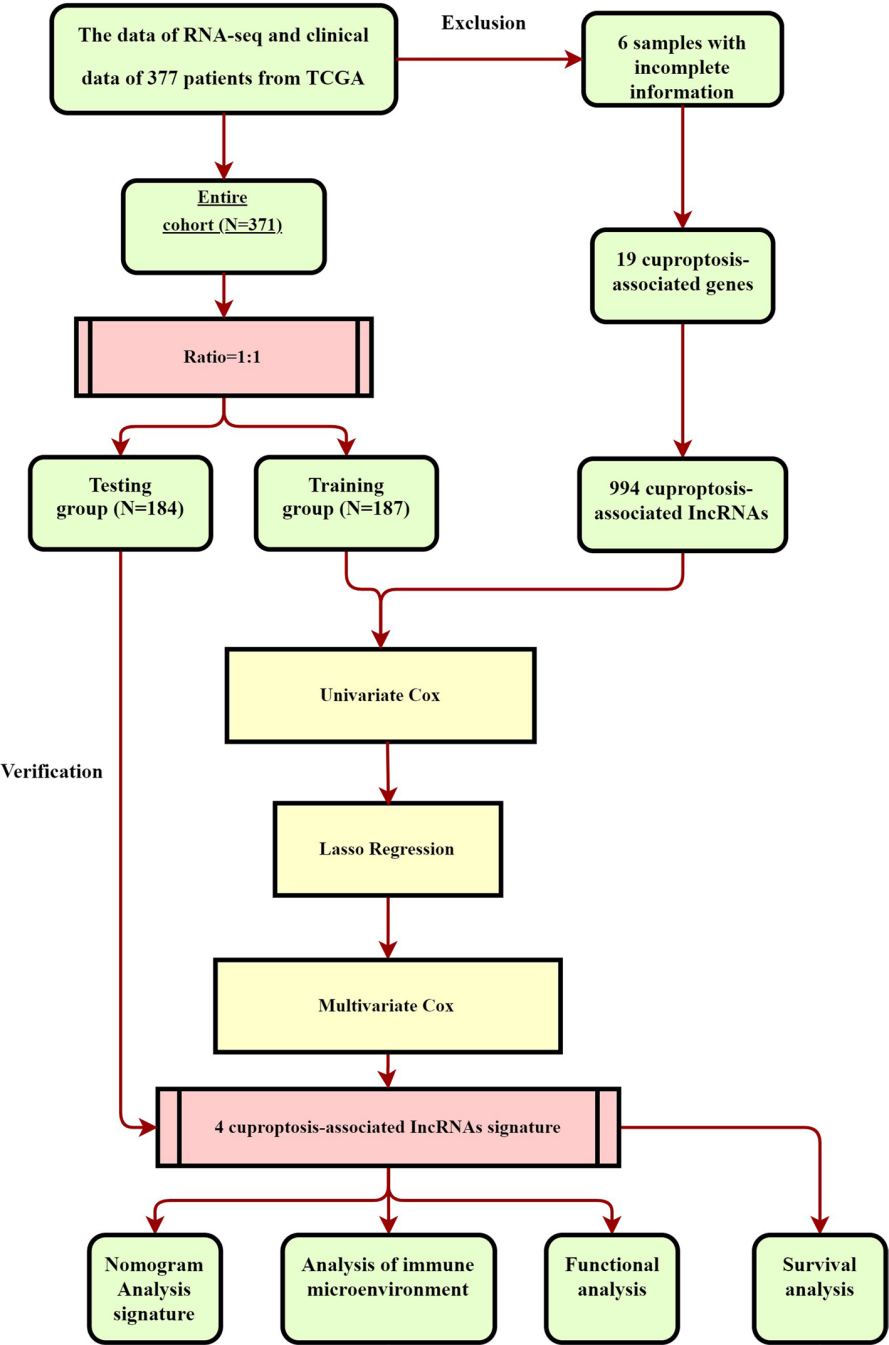
Overview of the study flow chart

We acquired the expression patterns for 374 LIHC samples and 50 corresponding healthy controls through the TCGA data. Utilizing annotation data acquired via the GENCODE webpage, we highlighted for further research 16,876 lncRNAs and 19,938 mRNAs. An expression matrix of 19 genes related to cuproptosis was retrieved through the TCGA-LIHC cohort. 996 lncRNAs associated with cuproptosis were identified for subsequent analysis. Figure 1 depicts the full research procedures.

### Construction and verification of the cuproptosis-related lncRNA predictive signature

**Fig. 2:**
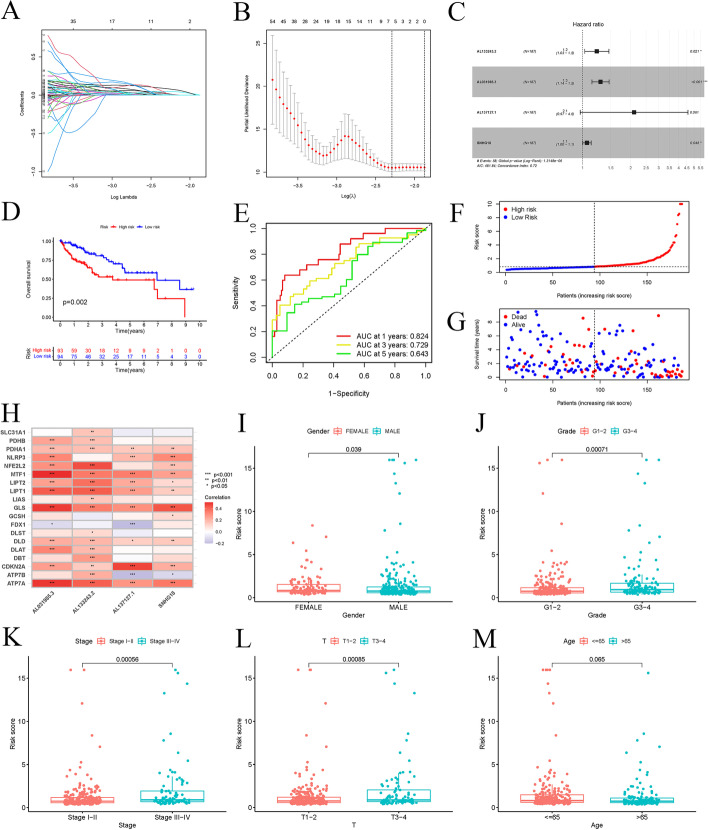
The construction of a prognostic signature in HCC patients. **a, b** According to minimum criteria, four cuproptosis-associated lncRNAs were selected by LASSO regression model; **c** coefficients; **d** K–M survival analysis showing that the high-risk group had poor prognosis and shorter overall survival in the training set; **e** ROC curve and AUCs at 1-, 3- and 5-year survival for the prognostic signature; **f, g** the scatter plot of risk scores and survival status in the training set; **h** the correlations between cuproptosis-associated genes and the four prognostic cuproptosis-associated lncRNAs in the proposed signature; **i–m**: risk and clinical correlation analysis (grade, gender, stage, T stage, and age are related to the risk score, P < 0.05)

For developing a predictive signature of lncRNAs linked to OS in LIHC patients, we random categorized TCGA-LIHC into training and testing sets (Additional file 1: Tables S2, S3). For the training set, in the univariate Cox regression assessment, we discovered 288 cuproptosis-related lncRNAs having predictive importance. Furthermore, multicollinearity was reduced by LASSO Cox regression, and four cuproptosis-related lncRNAs were selected (Fig. 2a–c). Finally, we created a prognostic signature including four cuproptosis-related lncRNAs (*AL133243.2*, *AL031985.3*, *AL137127.1*, *SNHG18*) (Fig. 2c). The risk score was measured as follows (Table 1): riskScore = (0.205 × AL133243.2_expression_) + (0.258 × AL031985.3_expression_) + (0.744 × AL137127.1_expression_) + (0.062 × SNHG18_expression_). In addition, the four genes responsible for creating the model were all significantly expressed in the high-risk group, according to our findings (Additional file 1: Figs. S1 and S2). There was a connection between cuproptosis-related genes and these four lncRNAs. *AL133243.2*, for instance, was positively connected with *NFE2L2*, *LIPT1*, and *ATP7A*, but adversely associated with *GCSH*. *AL031985.3* was linked positively to *MTF1*, *LIPT1*, and *GLS*, and adversely associated with *FDX1* (Fig. 2h).

**Table 1:** Prognostic signature genes identified from the multivariate Cox regression analysis

LncRNAs	HR (95% CI)	P-value	Coef
AL133243.2	1.228 (1.031–1.462)	0.021	0.205
AL031985.3	1.295 (1.144–1.466)	4.33E−05	0.258
AL137127.1	2.104 (0.965–4.586)	0.061	0.744
SNHG18	1.064 (1.001–1.132)	0.046	0.062

*HR*  hazard ratio, *CI* confidence interval, *Coef*  regression coefficient

Based on the median risk score, we classified the LIHC subjects in the training set into low- and high-risk categories. The K–M analysis of survival revealed that LIHC subjects having high-risk scores exhibited shorter OS as well as much poorer prognoses (Fig. 2d). Figure 2f, g depict the risk status and survival rates for every patient in the training set. In the first year, the AUC of the training cohort was 0.824, in the third year it was 0.729, and in the fifth year it was 0.656. (Fig. 2e). To determine the accuracy of the cuproptosis-related lncRNA prognostic signature, we connected clinical characteristics with the risk score (Fig. 2i–m). According to the data, tumor grade, age, T stage, and sex were significantly associated with a risk score.

**Fig. 3:**
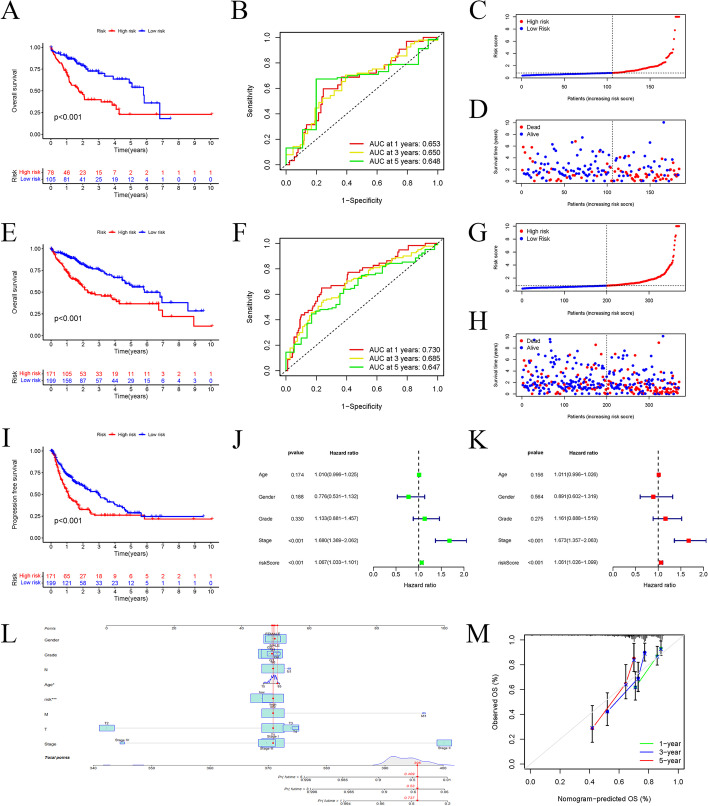
Internal validation of the prognostic signature and construction of the nomogram. **a** K–M survival curve in the testing cohort; **b** ROC curves and AUCs at 1-, 3-, and 5-year survival in the testing cohort; **c, d** the scatter plot of risk scores and survival status in the testing set; **e** K–M survival curve in the entire cohort; **f** ROC curve and AUCs at 1-year, 3-years and 5-years survival in the entire cohort; **g, h** the scatter plot of risk scores and survival status in the entire set; **i** evaluation of the predictive value of the cuproptosis-related lncRNA signature for PFS; **j** forest plot for univariate Cox regression analysis; **k** Forest plot for multivariate Cox regression analysis; **l** a hybrid nomogram based on cuproptosis-associated lncRNA signature for OS of HCC patients; **m** calibration curves of the nomogram to predict the 1-, 3-, and 5-year OS of HCC in all patients

For evaluating the predicting reliability of the model, we designed the survival curves for each of the testing and overall data (Fig. 3a, e). Risk status and survival outcomes for every patient in the testing set and all TCGA-LIHC patients are shown in Fig. 3c, d, g, h. AUC measures for OS for each of the datasets were > 0.6, demonstrating that the predicting modeling accurately forecasted the survival of HCC patients (Fig. 3b–f). Additionally, we analyzed the DFS of subjects in the low- and high-risk groups that declared that subjects in the high-risk group showed a shorter DFS contrasted with their low-risk counterparts (Fig. 3i). Univariate Cox regression and multivariate Cox regression examinations indicate that stage and risk score were significantly associated with OS in LIHC patients (Fig. 3j), and were irrespective determinants of OS in LIHC patients (Table 2, Fig. 3k). Regarding the challenging medical use of cuproptosis-related lncRNA risk score in estimating OS in LIHC patients, we merged it with clinicopathological parameters to develop a combined nomogram estimating 1-, 3-, and 5-year OS (Fig. 3l). Therefore, we created a nomogram containing gender, age, stage, T, N, M, and grade, as well as risk score predictors. The hypothesized model works identically to the ideal model, as demonstrated by the following standardization graphs (Fig. 3m).

**Table 2:** Univariate and multivariate Cox regression analysis of OS in TCGA-LIHC

Clinicopathologic parameters	Univariate analysis		Multivariate analysis	
	HR (95%CI)	*P*	HR (95%CI)	*P*
Age	1.010 (0.996–1.025)	0.174		
Gender	0.776 (0.531–1.132)	0.188		
Tumor grade	1.133 (0.881–1.457)	0.330		
Pathologic Stage	1.680 (1.369–2.062)	< 0.001*	1.673 (1.357–2.036)	< 0.001*
Risk score	1.067 (1.033–1.101)	< 0.001*	1.061 (1.026–1.099)	< 0.001*

*TNM* tumor-node-metastasis, *HR* hazard ratio

*P < 0.05 was considered statistically significant

**Fig. 4:**
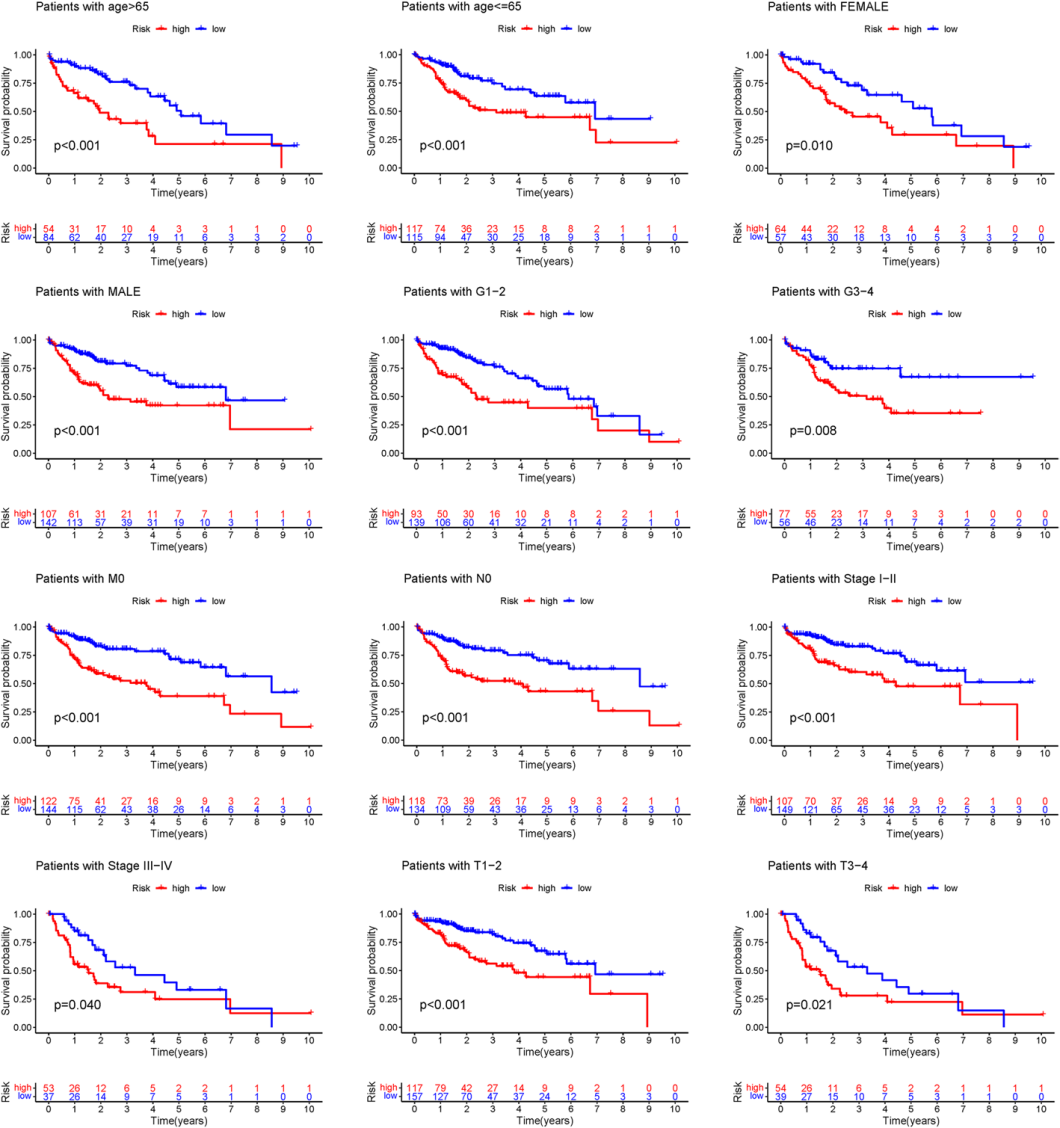
Survival curves for model validation. Our model could be applied to different clinical groups: age, gender, lymph node metastasis, grade, M stage, N stage, and T stage, P < 0.05

We also explored the association between the predictive parameters and OS of LIHC patients in the low- and high-risk groups, classified according to multiple clinicopathological variables such as age, gender, stage, T stage, and N stage. Subjects in the high-risk group showed significantly shorter OS for all of the key clinical features in comparison to the low-risk group (Fig. 4). Irrespective of clinicopathological factors, our findings indicate that predicting attributes can estimate the prognosis of LIHC patients. These four cuproptosis-related lncRNAs signature can successfully predict OS in LIHC patients.

### Tumor immune microenvironment of LIHC

**Fig. 5:**
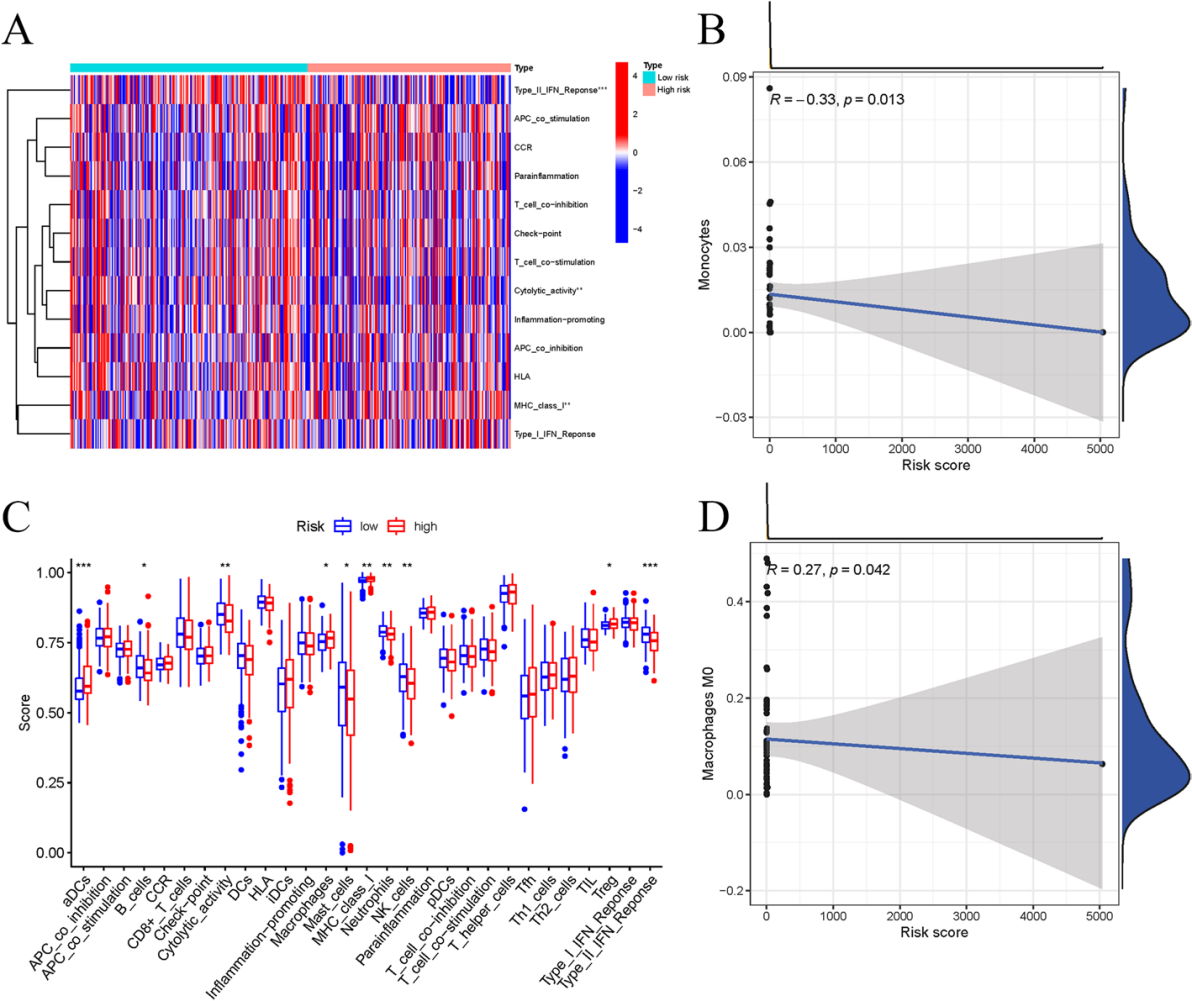
Comparison of immune infiltrating cells between high- and low-risk groups. **a, c** The scores of immune infiltrating cells and immune-related functions in high- and low-risk groups; The risk score was negatively correlated with the abundance of monocytes (**b**), while it was positively correlated with the abundance of macrophages M0 cells (**d**)

**Fig. 6:**
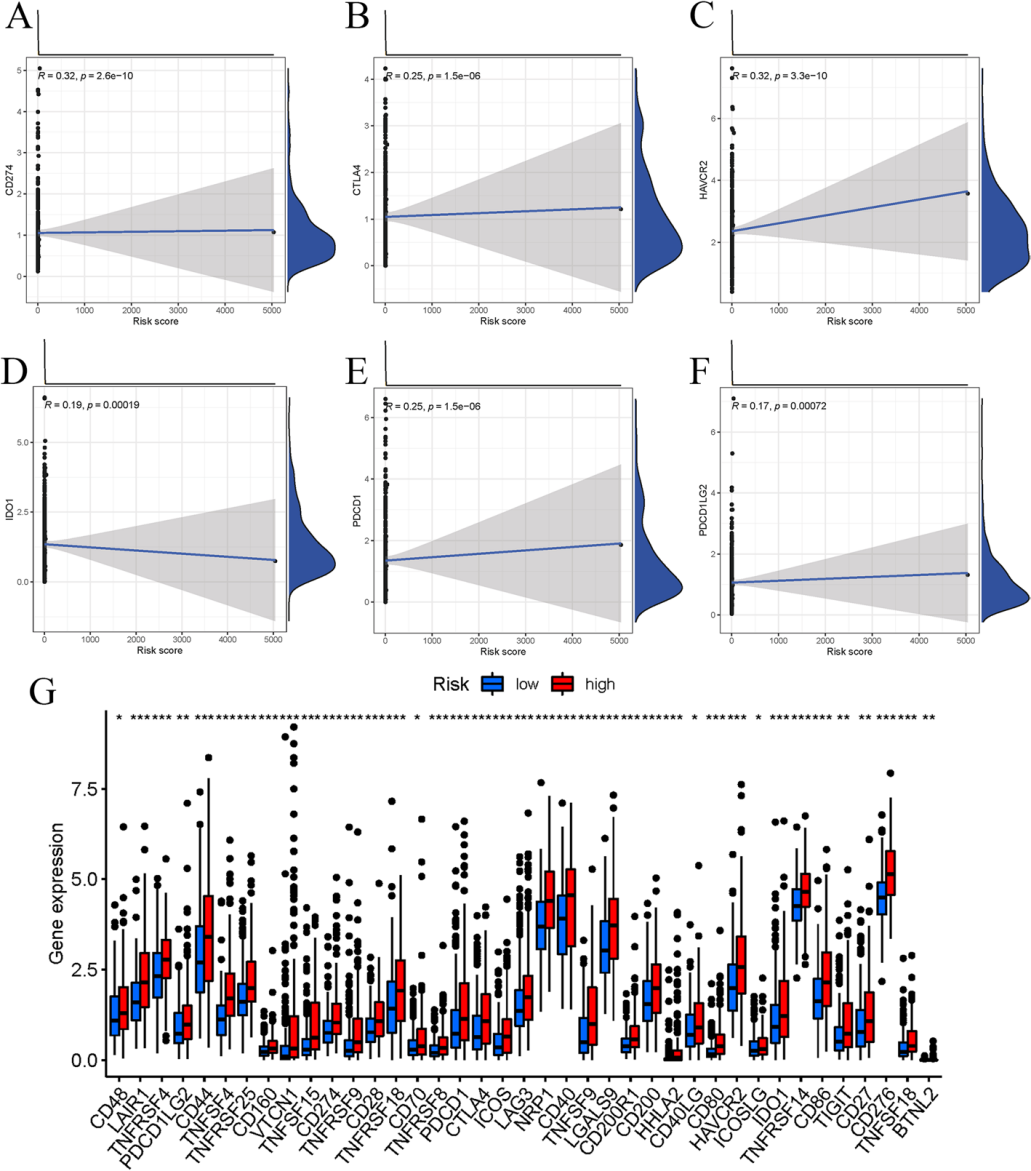
The analysis of the immune checkpoint genes between high-risk and low-risk groups and their correlation with the risk score. **a–f** Scatter plots of correlation analysis of risk score with PD-L1 and immune checkpoint genes infiltration level; **g** expression of immune checkpoint genes in high-risk and low-risk groups

TIME is an important element in carcinogenesis and cancer development. Its diversity influences patient prognosis and immunotherapy responsiveness [27]. To study the link between risk scores and the tumor immunological microenvironment across all patients, we utilized the CIBERSORT and XCELL algorithms to compute the proportions of various tumor-infiltrating immunity cells and the link between risk score and immunological cell density was determined (Fig. 5a). The risk score was significantly connected with the quantity of M0 macrophages and inversely related to monocytes, based on the scatter plots (Fig. 5b–d). Immune-related functionalities, like Type-II IFN reaction and cytolytic activity, were significantly greater prevalent in the low-risk group, as determined by a correlation study utilizing the GSVA and ssGSEA programs (Fig. 5c). Comparing the immunological checkpoint molecules between the two groups, we found that nearly all immunological checkpoint genes were up-regulated in the high-risk group (Fig. 6g). For accomplishing that, we examined the connection between the expression patterns of immunological checkpoint genes and the risk score. According to Fig. 6a–f, *PD-L1*, *CTLA4*, *HAVCR2*, *IDO1*, *PDCD1*, and *PDCD1LG2* were significantly correlated with the risk score.

**Fig. 7:**
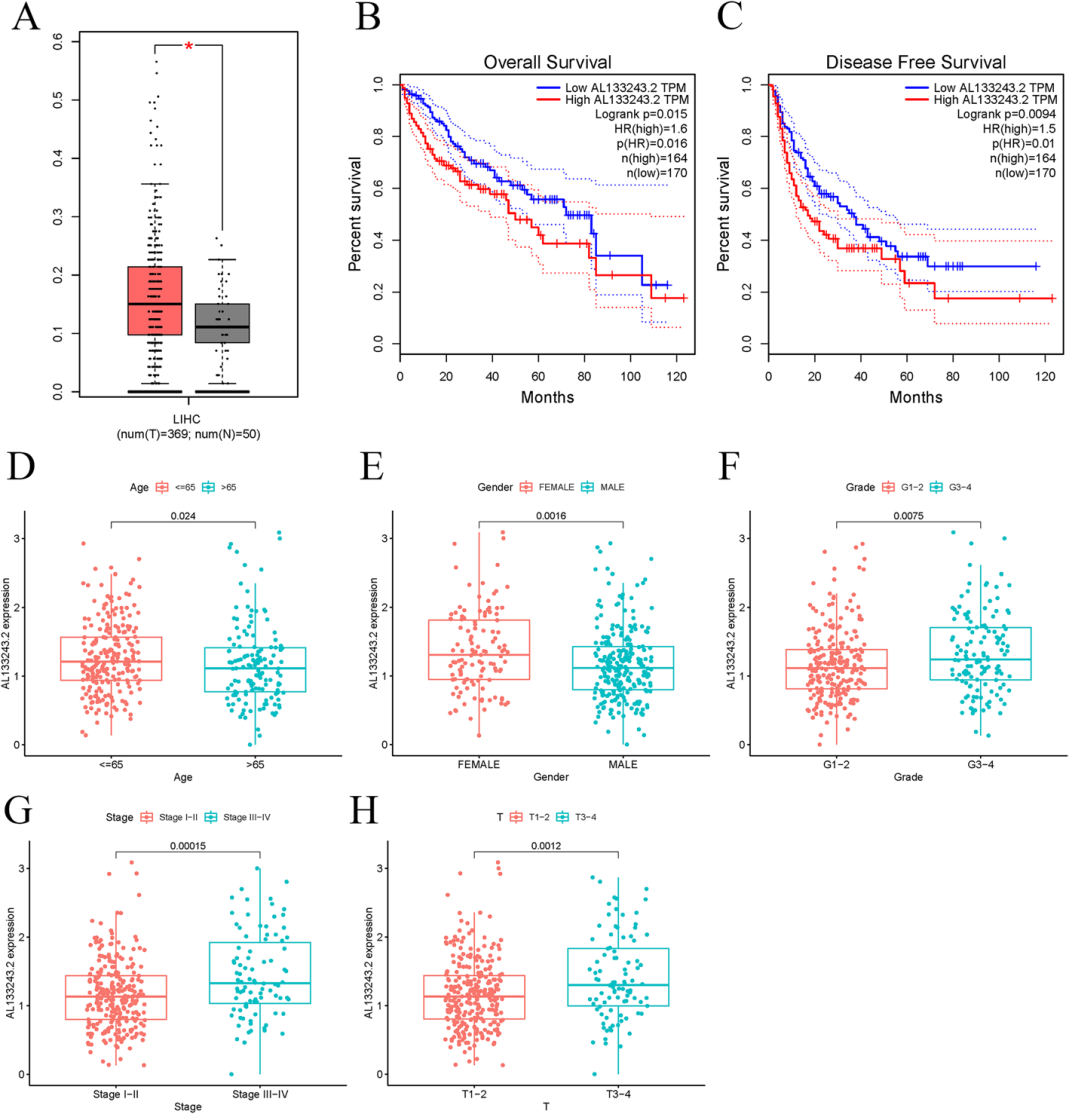
AL133243.2 expression and clinical correlation analysis. **a** Evaluating AL133243.2 in the GEPIA database; b, c OS plot and DFS plots from GEPIA database; **d–h** association between AL133243.2 expression and other clinicopathological features (AL133243.2 was highly expressed in female, G3-4, stages III-IV, T3-4, and patients under 65 years old, p < 0.05)

**Fig. 8:**
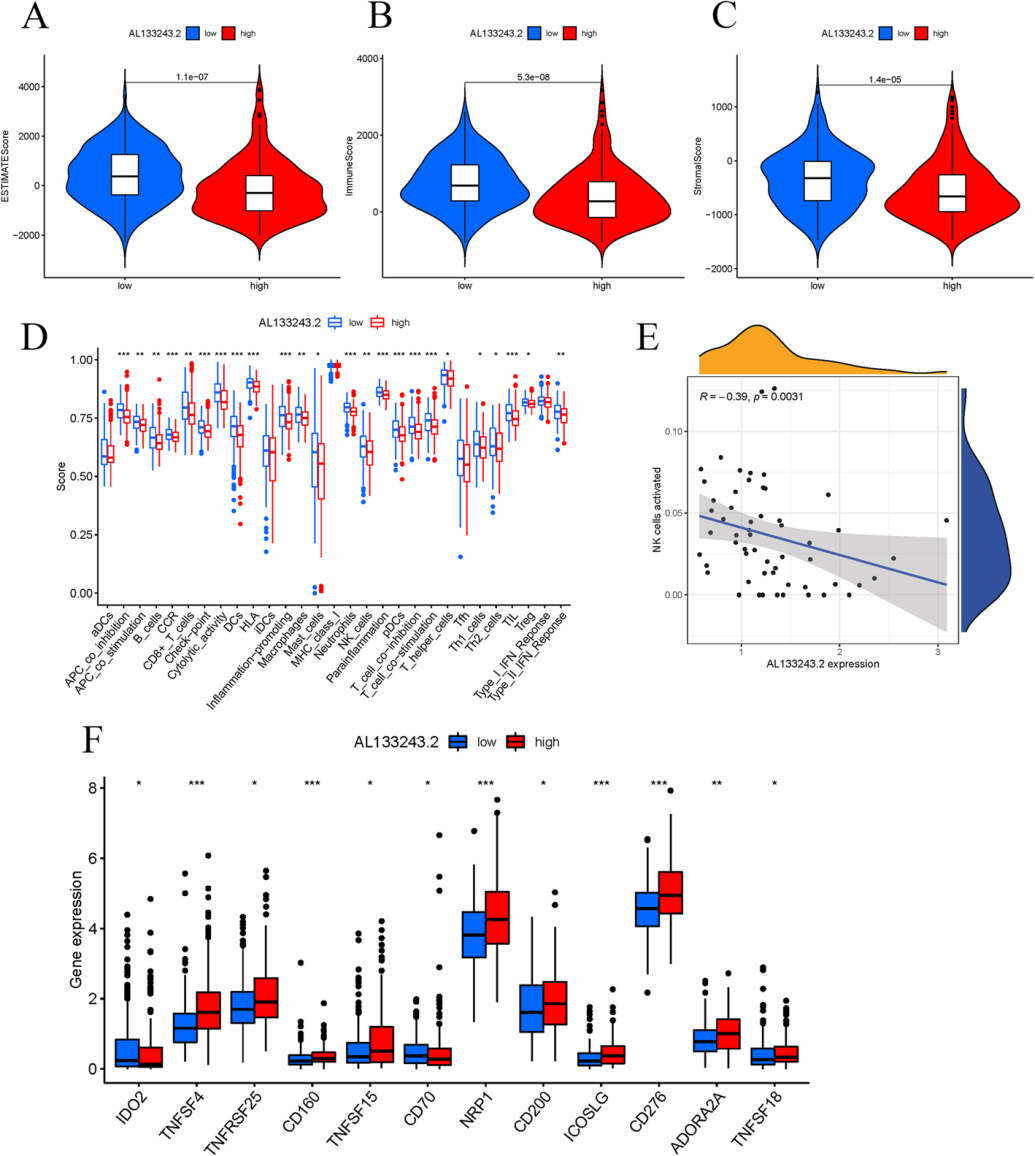
Difference analysis of tumor microenvironment, immune checkpoint genes expression level, and immune infiltration in different AL133243.2 expression groups. **a–c** All scores are higher in the high AL133243.2 expression group, indicating lower purity of tumors; **d, e** AL133243.2 expression and immune infiltration correlation analysis, activated NK cells were negatively related with the AL133243.2 expression, R < 0 and P < 0.05; **f** differences analysis in the expression of immune checkpoint genes between high and low AL133243.2 expression groups

For better exploration of the preventive and risk genes in the cuproptosis-related lncRNA predictive signature, we examined the connection between clinical parameters and gene expressions. *AL133243.2* was highly expressed, while *SNHG18* was expressed to a lower extent in the high-risk group. Using the GEPIA database, we then analyzed the connection between *AL133243.2*expression levels with OS or DFS. According to Fig. 7a, the expression of *AL133243.2* was raised in LIHC tumor tissues contrasted with the surrounding healthy tissues. A high expression of *AL133243.2* was also linked to shorter OS and DFS (Fig. 7b, c). For evaluating the connection between *AL133243.2* as well as clinical parameters, LIHC patients were splited into the *AL133243.2*^high^ and *AL133243.2*^low^ groups, as per the median expression pattern of *AL133243.2*. *AL133243.2* was highly expressed in grades 3–4 (Fig. 7f), T grade 3–4 (Fig. 7h), and stage III-IV tumors (Fig. 7g). Furthermore, *AL133243.2* expression was raised in patients under 65 and females (Fig. 7d, e). Subsequently, we applied ESTIMATE to calculate the stromal, immune, and ESTIMATE scores of *AL133243.2* groups with various expression levels. Furthermore, the stromal score, immune score, and ESTIMATE score were raised in the *AL133243.2*^low^ group contrasted with the *AL133243.2*^high^ group (Fig. 8a–c). Additionally, we also found that the infiltration ratios of activated NK cells were negatively correlated with the expression level of *AL133243.2* (R > 0 and P < 0.05, Fig. 8e). As shown in Fig. 8d, an elevation in *AL133243.2* expression was linked to a reduced level of the immunological cell infiltrate and higher expression of multiple immunological checkpoint genes in HCC (Fig. 8f). Therefore, *AL133243.2*^low^patients may be more responsive to immunological checkpoint treatment. The findings indicate that the *AL133243.2*^low^ LIHC patients may gain from immunotherapy better.

### Correlation between the predictive signature and LIHC therapy efficacy

**Fig. 9:**
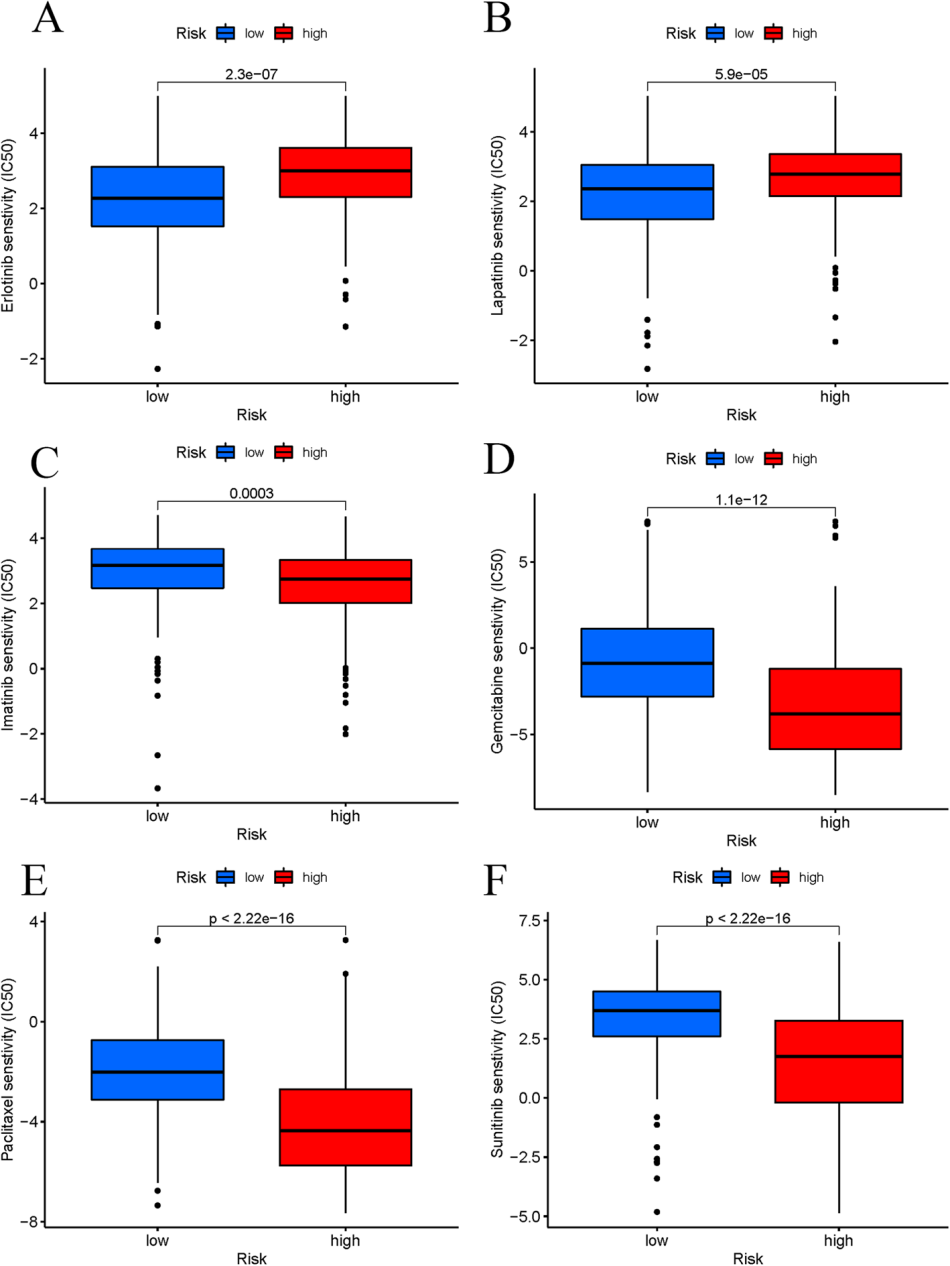
Chemotherapy drug sensitivity analysis in low- and high-risk groups. **a** Erlotinib; **b** lapatinib; **c** imatinib; **d** gemcitabine; **e** paclitaxel; **f** sunitinib

We also explored the connection between the predicting features of LIHC and chemotherapy effectiveness. These findings demonstrate that the IC50s of sunitinib, paclitaxel, imatinib, and gemcitabine were reduced in the high-risk group. In comparison, the IC50s of erlotinib and lapatinib were reduced in the low-risk group; this helps explore individualized treatment options for both the high- and low-risk groups (Fig. 9a–f).

### Functional enrichment and GSEA

**Fig. 10:**
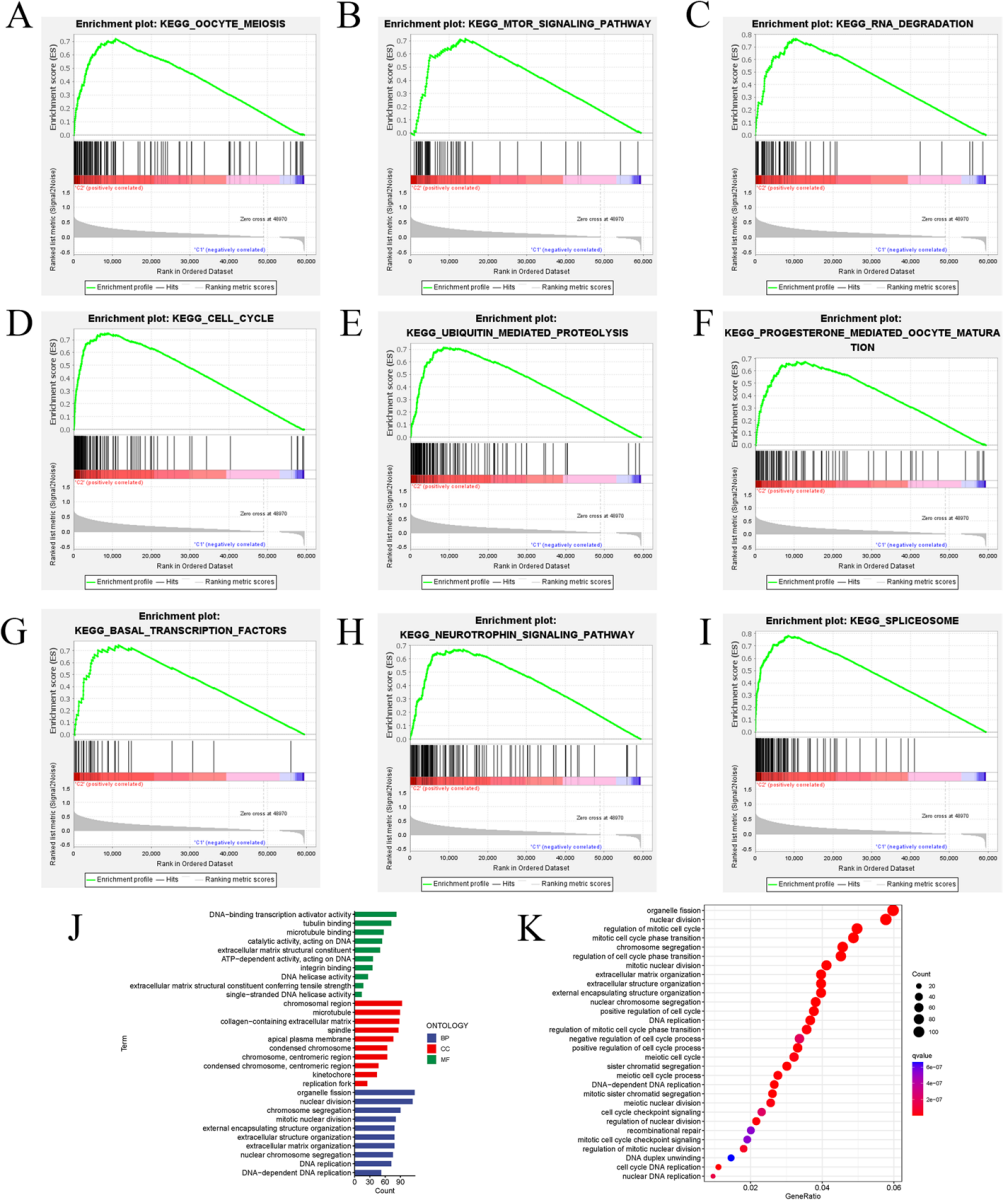
Gene function enrichment and signal pathway analysis. **a–i** Gene set enrichment analysis (GSEA) revealed that tumor hall markers were enriched in the high-risk group. All of them are positively related to C2. FDR < 0.05; **j** GO analysis; **k** KEGG analysis

Using R language and the set screening criteria, we obtained 2,497 DEGs from high and low risk groups. Through gene function enrichment and pathway analysis, GO results showed that significantly enriched functions relating to molecular functions (MF) were DNA binding transcription activator activity, tubulin binding, and microtubule binding; relating to cellular component (CC) were chromosomal region, microtubule, and collagen-containing extracellular matrix; relating to biological processes (BP) were organelle fission, nuclear division, and chromosome segregation. The KEGG pathway analyses indicated that DEGs were enriched in organelle fission, nuclear division, regulation of mitotic cell cycle, and chromosome segregation (Fig. 10j, k) [28, 29]. GSEA identified the tumor hallmark gene sets enriched in the high-risk group, including oocyte meiosis signaling, mTOR signaling pathway, RNA degradation, and cell cycle (Fig. 10a–i). These results offer novel perspectives into the molecular biological functions of cuproptosis-related lncRNAs.

## Discussion

HCC is the predominant histological subtype of primary liver malignancies. Worldwide, the prevalence of HCC fluctuates, although higher than 80% of instances happen in low- and middle-resources nations, often in East Asia and Africa [30–32]. Nevertheless, prompt detection and management of HCC continue to be a challenge, particularly in underdeveloped nations with poor healthcare [33]. Even after significant increases in survival following the extensive multidisciplinary intervention, such as surgery, radiation, chemotherapy, and immunotherapy, about 70% of HCCs recur within five years post resection or ablation, with a poor prognosis [34, 35]. Currently, The majority of patients are initially spotted within the middle and late stages due to insignificant clinical symptoms in the early stages, thus missing the optional treatment period [36, 37]. Therefore, identifying biomarkers for diagnosis and prognosis of HCC requires more effort, and their deployment is advantageous for the efficient and prompt therapy of patients.

RCD plays a crucial involvement in several pathological and physiological activities, involving carcinogenesis [38]. Tsvetkov et al. recently reported that cuproprosis is a unique sort of programmed cell death having double roles in tumorous establishment and therapy. Consequently, understanding the biological process of cuproptosis in tumor cells may provide novel insights into new therapeutic targets [9]. Correlations between cuproptosis regulators and lncRNA signatures were examined in several cancers, just like melanoma [11]. Tumor patients were categorized into subgroups based on their molecular attributes, including gene signatures of cuproptosis-related lncRNAs. This allows for predicting their different phenotypes, treatment responses, and prognoses [12]. As a type of RCD, the function of cuproptosis in HCC is still unclear.

In recent years, with the development of high-throughput data, microarray data processing has become one of the important applications of molecular biology in cancer diagnosis [39–41]. In this research, for the first time, we assessed the predictive importance of cuproptosis-related lncRNA gene signatures in HCC. We discovered that 288 cuprotosis-related lncRNA genes were significantly linked to OS in HCC patients. We constructed a prognostic signature containing four cuproptosis-related lncRNA genes through LASSO regression and multivariate Cox regression testing. By the median value of prognostic characteristics, HCC patients were splited into high- or low-risk groups. It was observed that subjects in the former group exhibited a longer OS duration contrasted with the latter. We found that the four cuproptosis-related lncRNAs that were modeled were all up-regulated in the high-risk group. Through high-throughput bioinformatics analysis, many studies have shown that *AL031985.3* is associated with the prognosis of HCC and can be used to build a predictive model for the prognosis of HCC [42–44]. Previous studies have found that SNHG18 is abnormally expressed in glioma tissue specimens, and high expression of SNHG18 in gliomas enhances their radiation resistance [45–47].

To further explore the potential mechanism of cuproptosis related lncRNA genes affecting the prognosis of HCC. We analyzed its role in immune infiltration and evaluated immune cell scores in the high- and low-risk groups.The results demonstrate that macrophage M0 was significantly linked to the risk score, whereas the infiltration ratio of monocyte cells was adversely linked to this parameter. Macrophages are an old cell kind contributed to tissue homeostasis and pathogen fighting [48]. Tumor-associated macrophages (TAMs) in the tumor microenvironment are heterogeneous subsets, including M1 antitumor and M2 tumor-promoting phenotypes. TAMs primarily exert M2-like tumor-promoting effects in the TME and regulate various malignant effects, such as angiogenesis, immunosuppression, and tumor metastasis [49, 50]. Prior work has reported that each of the M1 and M2 macrophages are produced by M0 is a quiescent state of macrophages with no particular role until they become polarized [51]. M0-like macrophages are linked to high-grade tumors and a bad fate in gliomas [52, 53]. This research detected that vast immunological checkpoint genes were up-regulated in LIHC tissues in the high-risk group. High-risk HCC subjects exhibited a bad fate due to increased tumor immunological avoidance.

After GEPIA database evaluation, we discovered that *AL133243.2* was substantially expressed in LIHC cancerous tissue relative to neighboring healthy tissue, and its expression was connected with a poorer DFS and OS. Furthermore, *AL133243.2* overexpression within the tumor tissues was positively linked to multiple immune checkpoint gene expression. Additionally, *AL133243.2* expression was raised in grades 3–4 and staged III- IV, as well as in patients under 65. These findings suggest that *AL133243.2* has a salient role in LIHC progression, although its functionality is unclear. Previous findings found that high expression of *AL133243.2* was closely associated with poor prognosis in patients with endometrial cancer [54, 55]. We also observed that the infiltration ratio of activated NK cells was adversely linked to expression pattern; a greater expression of *AL133243.2* was correlated with a reduced degree of immunological cell infiltration in HCC. NK cells fall under the cell type of the innate lymphoid branch. Tumorous immunological infiltrate, especially tumor-infiltrating lymphocytes, is linked to better survival in the majority of solid cancers [56–58]. It is generally assumed that low levels of NK cell infiltration and NK cell dysfunction are connected with worse OS as well as tumor recurrence following therapy [58, 59]. Therefore, cuproptosis-related lncRNAs can impact the fate of HCC patients by affecting tumor immune infiltration.

Furthermore, for investigating the possible cuproptosis-related lncRNA pathways in the establishment of LIHC, we annotated the cuproptosis-related lncRNAs through GO and KEGG tests in a practical way. The results demonstrate that it is primarily connected with the regulatory function of the cell cycle. KEGG analysis further demonstrates that the organelle fission, nuclear division, regulation of the mitotic cell cycle, and chromosome segregation signaling pathways were enriched. Therefore, cuproptosis-related lncRNAs can impact the fate of LIHC patients by regulating cell cycle-related functions, but their roles and mechanisms in LIHC have not yet been reported. GSEA also identified oocyte meiosis signaling, and mTOR signaling pathways were enriched. The results show that cuproptosis-related lncRNAs are closely associated with oocyte meiotic DNA replication and cell division, which is identical to the quick growth and division of tumor cells [60]. Malignant development, which is the defining hallmark of cancer, is caused when the cell cycle is allowed to progress out of control. In many cases, the aggressive and unintentional spread of cancer is caused by erroneous phenotypes that result from DNA replication [61]. It is possible to effectively analyze the PI3K/AKT/mTOR signaling pathway as an appropriate treatment option because it is connected with several elements of cell growth and survival and is commonly activated in many different types of cancer [62]. The PI3K/AKT/mTOR axis can exhibit oncogenic activity by blocking autophagy and senescence processes while concurrently promoting cell growth, survival, migration, and invasiveness [63, 64]. Given that the signaling pathways involved in the LIHC process are under-investigated, the findings offer vital perspectives that can be used for new paths in the creation of innovative diagnostic and medication techniques.

In conclusion, cuproptosis-related lncRNAs can promote the aggressiveness and antiapoptotic abilities of liver cancer cells through the PI3K/AKT/mTOR pathway, thus affecting liver cancer tumor progression. Therefore, cuproptosis-related lncRNAs may be a viable predictive indicator and an effective target for therapeutic intervention in LIHC. Notably, This investigation includes many restrictions. First, although machine learning and computational biology have developed to a certain extent, the computational complexity of gene selection is too high, so the efficiency of screening genes is low, especially in the process of drug sensitivity analysis. Secondly, in the process of screening prognostic genes to build a risk prediction model, due to the large amount of gene data and the relationship between protein interactions, the genes of the constructed model are different in each run, which leads to the possibility that the constructed model is not optimal, and it also increases the difficulty of screening the key genes of the disease. Thirdly, a large number of studies have shown that the methods of gene selection and model optimization in machine learning have been improved [65–67]. However, in medicine, it is not enough to rely on the analysis of big data alone, and further verification in vitro is needed. In addition, with the rapid growth of molecular and clinical data, data processing and analysis will meet greater challenges [65, 68]. Especially in medicine, the processing of data and the screening of disease-critical genes need to be more targeted.

## Conclusions

We proposed a predictive cuproptosis-related lncRNA signature model for LIHC, in addition to a predictive nomogram according to the 4-gene risk score and age of patients. Overexpression of *AL133243.2* was particularly linked to a much poor prognosis, raised levels of immune checkpoint genes, and raised immunological cell infiltration. Therefore, LIHC patients having high levels of *AL133243.2* in tumors may respond better to immunotherapies such as immunological checkpoint inhibition.

## Supplementary Information

**Additional file 1**. 19 cuproptosis-related lncRNAs, basic clinical data of LIHC patients in training set and test set, and the expression of 4 cuproptosis-related lncRNAs in the training set and test set in the model.

## Data Availability

At TCGA, the dataset utilized to support the study's results is accessible with a valid request. The article/Supplementary Material lists the name(s) of the repository(s) and the access number(s).

## Abbreviations

LIHC: Liver hepatocellular carcinoma
HCC: Hepatocellular carcinoma
TIME: Tumor immune microenvironment
TME: Tumor microenvironment
DFS: Disease-free survival
TCGA: The Cancer Genome Atlas
KEGG: Kyoto Encyclopedia of Genes and Genomes
GSEA: Gene Sets Enrichment Analysis
GO: Gene Oncology
OS: Overall survival
ICB: Immune checkpoint blockade
lncRNA: Long non-coding RNA
FDR: False discovery rate
