# Supplementary material for: Comprehensive analysis of cuproptosis-related lncRNAs in immune infiltration and prognosis in hepatocellular carcinoma
Source: BMC Bioinformatics. 2023 Jan 3;24:4. doi: 10.1186/s12859-022-05091-1 (PMC9811804; doi:10.1186/s12859-022-05091-1)

**Table S1. 19 cuproptosis-related genes from published literature.**

| **Genes** |  |
| --- | --- |
| NFE2L2 |  |
| NLRP3 |  |
| ATP7B |  |
| ATP7A |  |
| SLC31A1 |  |
| FDX1 |  |
| LIAS |  |
| LIPT1 |  |
| LIPT2 |  |
| DLD |  |
| DLAT |  |
| PDHA1 |  |
| PDHB |  |
| MTF1 |  |
| GLS |  |
| CDKN2A |  |
| DBT |  |
| GCSH |  |
| DLST |  |

**Table S2. Basic clinical information of 187 LIHC patients in the training set.**

| **Variables** | **Training set**  **(n=187)** |
| --- | --- |
| Age |  |
| <=65 | 116(62.03%) |
| >65 | 71(37.97%) |
| Gender  Female  Male | 58(31.02%)  129(68.98%) |
| Tumor Grade  G1&G2  G3&G4  Unknown | 118(63.1%)  67(38.83%)  2(1.07%) |
| Pathologic Stage  I&II  III&IV  Unknown | 124(66.31%)  53(28.34%)  10(5.35%) |
| AJCC-T  T1  T2  T3  T4  Unknown | 83(44.39%)  49(26.2%)  46(24.6%)  8(4.28%)  1(0.53%) |
| AJCC-N  N0  N1  Unknown | 132(70.59%)  4(2.14%)  51(27.27%) |
| AJCC-M  M0  M1  Unknown | 143(76.47%)  1(0.53%)  43(22.99%) |

**Table S3. Basic clinical information of 184 LIHC patients in the testing set.**

| **Variables** | **Testing set**  **(n=184)** |
| --- | --- |
| Age |  |
| <=65 | 116(63.04%) |
| >65 | 67(36.41%) |
| Unknown | 1(0.54%) |
| Gender  Female  Male | 63(34.24%)  121(65.76%) |
| Tumor Grade  G1&G2  G3&G4  Unknown | 114(61.95%)  67(36.41%)  3(1.63%) |
| Pathologic Stage  I&II  III&IV  Unknown | 133(72.29%)  37(20.11%)  14(7.61%) |
| AJCC-T  T1  T2  T3  T4  Unknown | 98(53.26%)  45(24.46%)  34(18.48%)  5(2.72%)  2(1.09%) |
| AJCC-N  N0  N1  Unknown | 120(65.22%)  0(0%)  64(34.78%) |
| AJCC-M  M0  M1  Unknown | 123(66.85%)  3(1.63%)  58(31.52%) |

**Figure S1.** Heatmap of four cuproptosis-related lncRNAs expression in the training dataset.


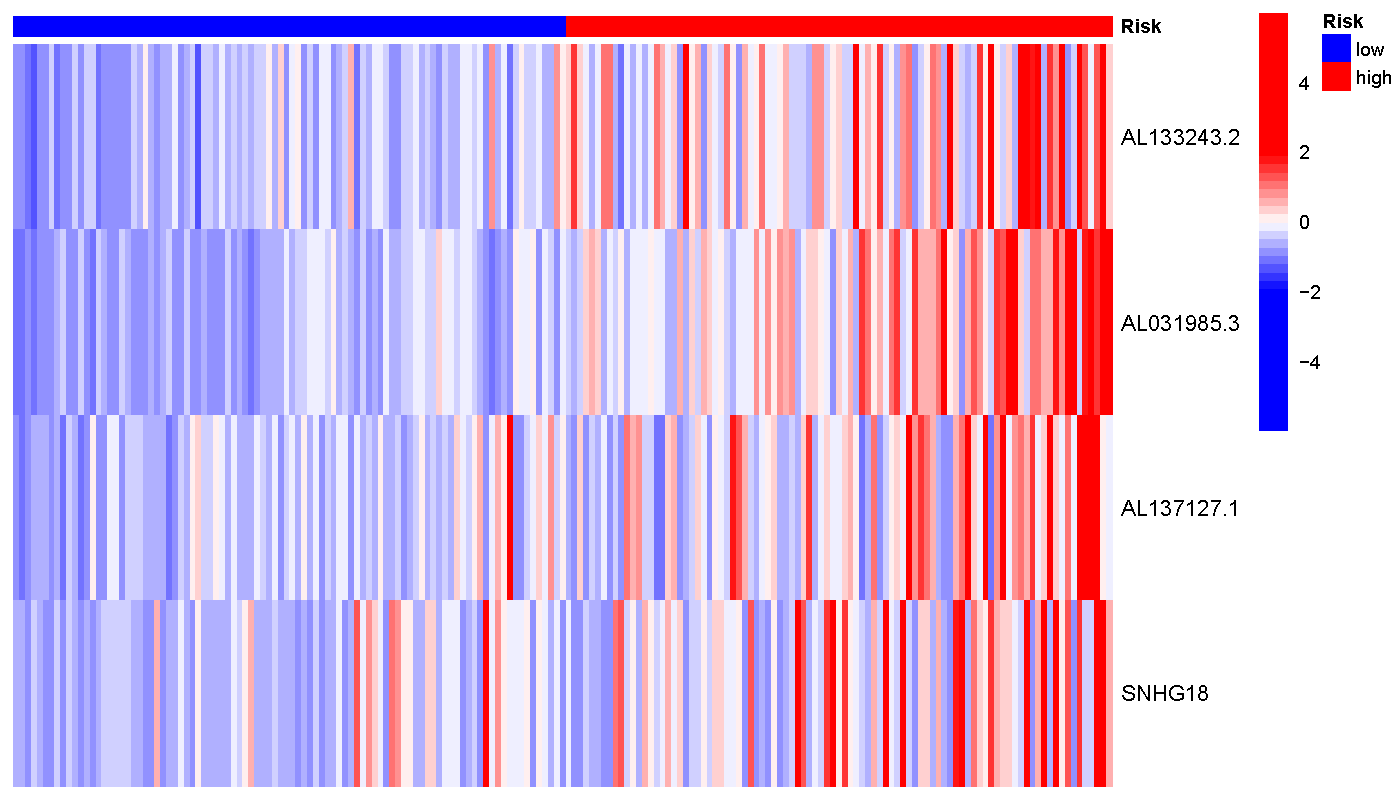


**Figure S2.** Heatmap of four cuproptosis-related lncRNAs expressions in the testing dataset.


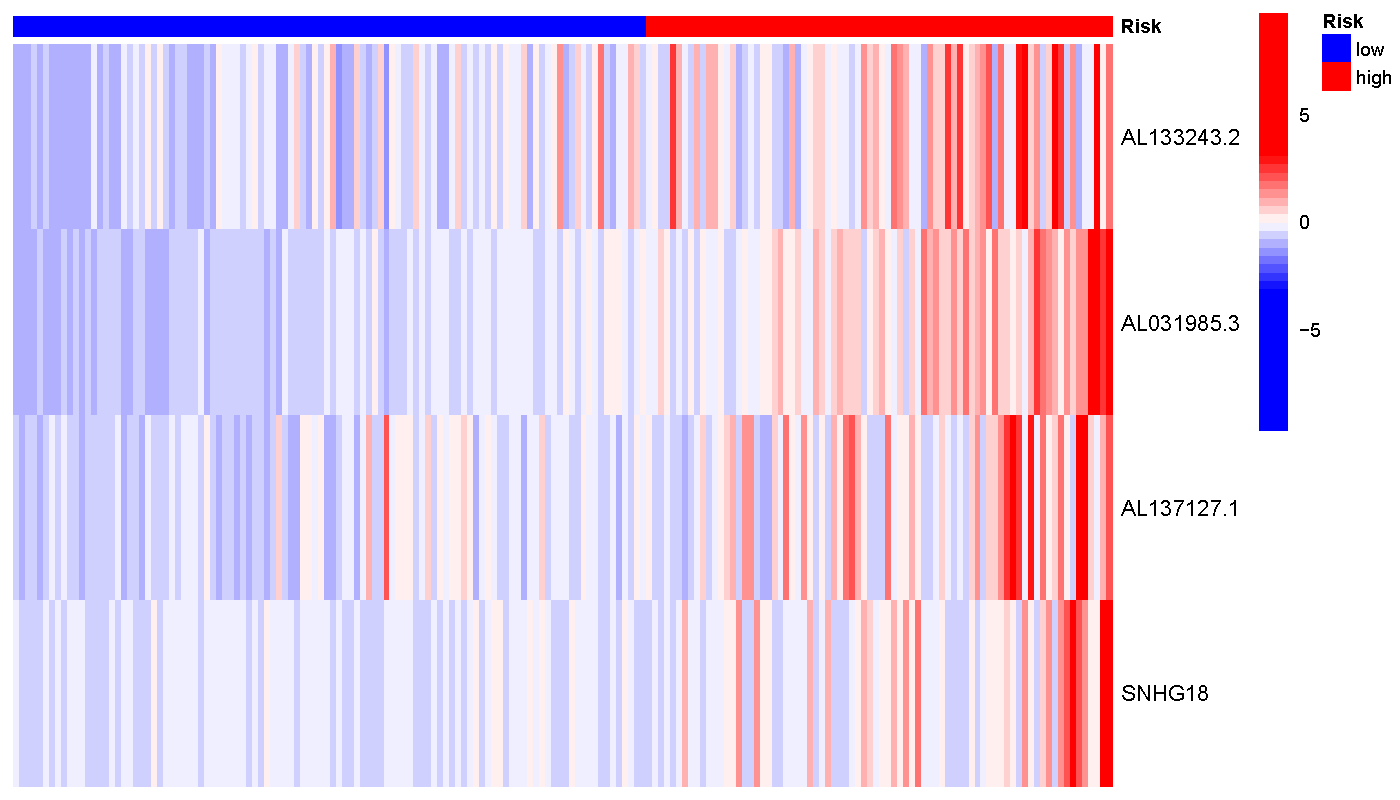

Supplement: Supplementary file 1 — Additional file 1. 19 cuproptosis-related lncRNAs, basic clinical data of LIHC patients in training set and test set, and the expression of 4 cuproptosis-related lncRNAs in the training set and test set in the model. [file 12859_2022_5091_MOESM1_ESM.docx]
